# A comprehensive review of the applications of bacteriophage-derived endolysins for foodborne bacterial pathogens and food safety: recent advances, challenges, and future perspective

**DOI:** 10.3389/fmicb.2023.1259210

**Published:** 2023-10-06

**Authors:** Fazal Mehmood Khan, Jie-Hua Chen, Rui Zhang, Bin Liu

**Affiliations:** ^1^College of Civil and Transportation Engineering, Shenzhen University, Shenzhen, China; ^2^Institute for Advanced Study, Shenzhen University, Shenzhen, China; ^3^Institute for Innovative Development of Food Industry, Shenzhen University, Shenzhen, China

**Keywords:** antimicrobial resistance, bacteriophage, endolysin, foodborne bacterial pathogens, food safety

## Abstract

Foodborne diseases are caused by food contaminated by pathogenic bacteria such as *Escherichia coli, Salmonella, Staphylococcus aureus, Listeria monocytogenes, Campylobacter,* and *Clostridium,* a critical threat to human health. As a novel antibacterial agent against foodborne pathogens, endolysins are peptidoglycan hydrolases encoded by bacteriophages that lyse bacterial cells by targeting their cell wall, notably in Gram-positive bacteria due to their naturally exposed peptidoglycan layer. These lytic enzymes have gained scientists’ interest in recent years due to their selectivity, mode of action, engineering potential, and lack of resistance mechanisms. The use of endolysins for food safety has undergone significant improvements, which are summarized and discussed in this review. Endolysins can remove bacterial biofilms of foodborne pathogens and their cell wall-binding domain can be employed as a tool for quick detection of foodborne pathogens. We explained the applications of endolysin for eliminating pathogenic bacteria in livestock and various food matrices, as well as the limitations and challenges in use as a dietary supplement. We also highlight the novel techniques of the development of engineering endolysin for targeting Gram-negative bacterial pathogens. In conclusion, endolysin is safe and effective against foodborne pathogens and has no adverse effect on human cells and beneficial microbiota. As a result, endolysin could be employed as a functional bio-preservative agent to improve food stability and safety and maintain the natural taste of food quality.

## 1. Introduction

Food-borne sickness and death are caused by bacterial pathogens such as *Escherichia coli*, *Salmonella, Staphylococcus aureus, Listeria monocytogenes, Campylobacter,* and *Clostridium*, which represent an increasing problem for both the food market and healthcare system on a global basis. According to the World Health Organization (WHO), an estimated six hundred million people become sick from consuming contaminated food, and approximately 420,000 die yearly from food-borne illnesses (Kulinkina et al., 2016). This has affected the global economy to US$110 billion (Kulinkina et al., 2016). Despite increased efforts to enhance hygiene practices and spread awareness, outbreaks of food-borne illnesses have not been diminished (Bintsis, 2017). Pathogenic bacteria can cause food-borne diseases and contamination at any step in the food storage and distribution chains, such as food production, transportation, handling, and packing. To sustain the safety of the food supply chain, effective interventions for foods and the food processing environment are required. Foodstuff processing companies use various methods, such as high-pressure processing (HPP) and radiation, to reduce bacterial load in food products. However, these methods have drawbacks. HPP can produce unpleasant sensory changes in some foods, as well as cause damage to the texture of fragile food. Irradiation could negatively impact the organoleptic properties of foods (Bajovic et al., 2012; Maherani et al., 2016; Wang C.-Y. et al., 2016). Different chemicals, including acetic acid, acetates, diacetates, lactates, dehydroacetic acid, chlorine, hypochlorite, peroxyacetic acid, sodium propionate, and acidified calcium sulfate, are used as antimicrobial agents in meat, dairy, and other food products. However these chemicals leave residues of harmful compounds in food, and their use in foods has low customer interest (Sohaib et al., 2016). Antibiotics are one of the livestock’s most common antimicrobial agents. Antibiotics are widely used in poultry to help stop disease outbreaks and treat illnesses, and they are also the most commonly used feed supplements to increase mammal growth (Mehdi et al., 2018). But antibiotics kill a broad range of bacterial strains, causing the disturbance of microbiota composition in food products, especially the substantial loss of beneficial bacteria (Endersen et al., 2014). In addition, this leads to the emergence of multidrug-resistant (MDR) bacteria, a critical threat to human health and the livestock industry (Boeckel et al., 2015; Ma et al., 2021).

The uses of these methods potentially reduce customers’ satisfaction and use. As a result, it is urgently necessary to develop novel and innovative techniques for controlling food-borne bacteria. The bacteriophage-derived endolysins, in particular, provide us with an excellent natural way of developing measures to prevent food-borne pathogens (Lee et al., 2022). Bacteriophages are essential biological self-reproducing organisms and are the specific killers of bacterial cells (Oyejobi et al., 2022; Mehmood Khan et al., 2023). Endolysins are bacteriophage-derived lytic proteins and have several benefits over other antibacterial compounds, such as a high degree of activity against antibiotic-resistant bacterial pathogens, rapid bacterial lysis, high specificity for the target bacterium, low resistance potential of bacterial-resistant mutants, and good efficacy in planktonic and biofilm cells (Abdelrahman et al., 2021; Gondil et al., 2021). Endolysins have gained recognition as antibacterial agents capable of being effectively used as natural preservatives in processing and packaging systems to limit pathogens and, ultimately, increase food safety. Endolysin treatment is a cutting-edge strategy for coping with the growing problem of antibiotic-resistant bacteria in the food industry (Chang, 2020). This review described the key features of endolysins, the synthetic engineering of endolysin, the applications of endolysins against food-borne bacteria and the current challenges and future perspectives on the development of endolysin for food safety.

### 1.1. Mechanism of action of the endolysin

**Figure 1:**
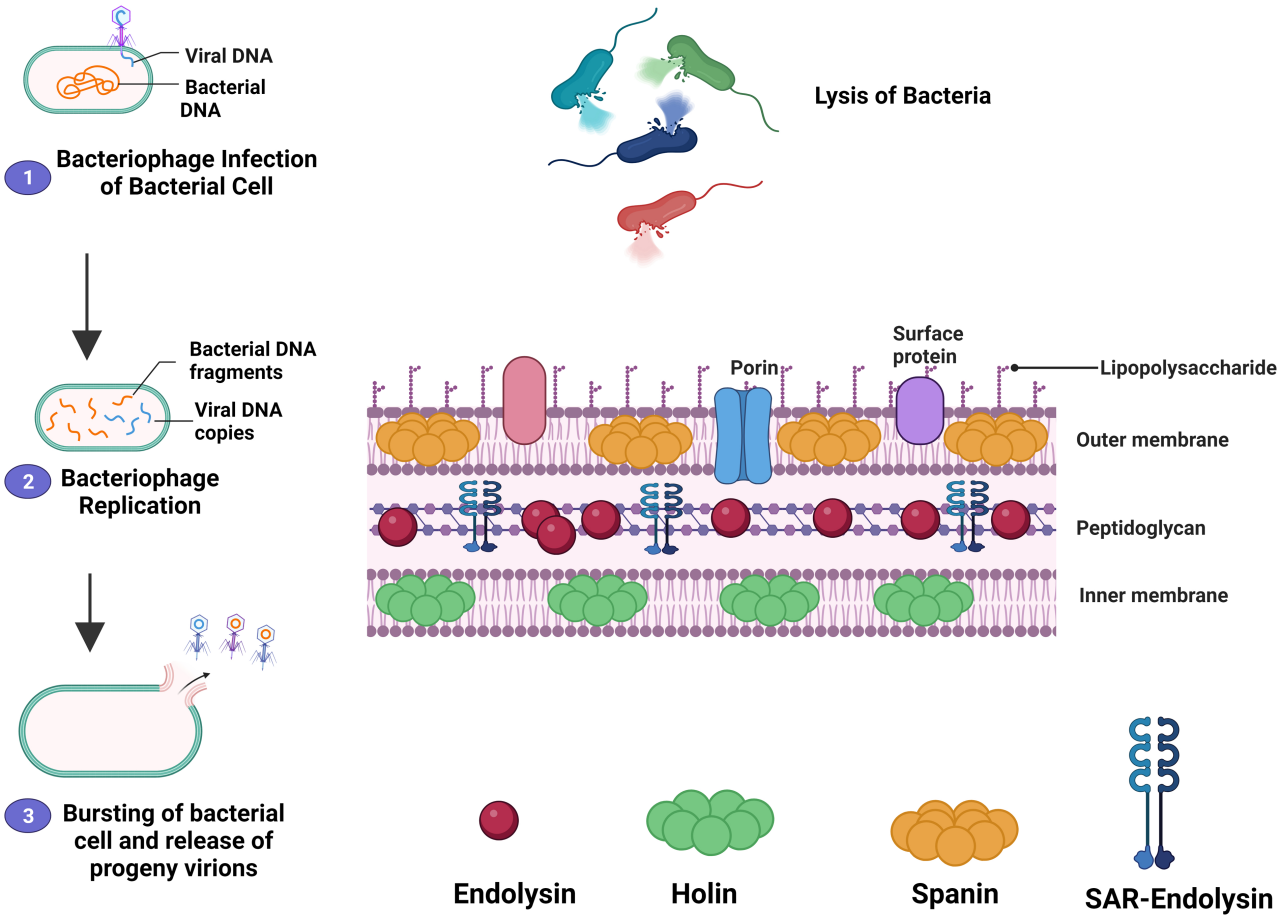
The mechanism of action of the lytic cycle of bacteriophage and bacteriophage-derived endolysin is described schematically. The figure shows how the holin-endolysin reaches and penetrates the Peptidoglycan layer and lysis the bacteria. The mechanism shows that the bacteriophage recognizes and attaches to bacterial-specific cell receptors through specific tail fiber protein in the first step. After attachment, the bacteriophage inserts its genetic material into the bacteria through the tail then it forms a progeny phage. The holin makes a channel for the entry and delivery of endolysin to the bacterial Peptidoglycan layer. In the last step, bacterial cell degradation occurs through endolysin.

There is essentially a three-step model system of bacterial host lysis. This model is made up of three proteins: endolysin, holin, and spanin. These proteins act on the bacterial outer membrane, inner membrane, and peptidoglycan, and can cause host lysis. In Gram-negative hosts, there are two different processes for peptidoglycan degradation: (1) Holin-endolysin and (2) Pinholin SAR endolysin. Degradation of the peptidoglycan is in the preceding stage when holin makes small pores in the host inner membrane; lysis is then initiated by releasing endolysin inside the periplasm and depleting the peptidoglycan. In the final phase, lysis begins when the pinholin may induce membrane depolarization, activating the secreted SAR endolysin. Previously, it was thought that degradation of the first two membrane barriers was sufficient for the lysis of Gram-negative hosts, but current research indicates that a third functional class of lysis proteins, spanins, is now believed to be essential for outer membrane disruption. Spanins get their name from the fact that they form a protein bridge that connects both membranes. Most phages create a two-component spanin complex, which is made up of an outer membrane lipoprotein (o-spanin) and an inner membrane protein (i-spanin) containing a coiled-coil periplasmic domain. With the N-terminal lipoprotein signal and a C-terminal transmembrane domain, some phages have a novel sort of spanin that spans the periplasm as a single molecule. Furthermore, the meshwork of the peptidoglycan inhibits spanin action, thus linking the spanin step to the first two processes mediated by holin and endolysin. These proteins can cause the bacterial host cell to lysis (Cahill and Young, 2019; Abdelrahman et al., 2021). Figure 1 illustrates the mechanism of action of endolysin.

### 1.2. Structure of endolysin

The majority of endolysins have a modular structure, whereas endolysins acting on Gram-negative bacteria have a basic globular structure (Oliveira et al., 2018). Modular endolysins are differentiated by the presence of one or two (multi-domain) N-terminal enzymatically active domain (EAD) linked to a C-terminal cell wall-binding domain (CBD) by a short, flexible linker region. Phages infecting Gram-positive bacteria and mycobacteria both possess such a structure (Oliveira et al., 2018; Gondil et al., 2021). The N-terminal EAD of modular endolysins cleaves specific peptidoglycan bonds in the host bacterium’s murein layer, whereas the C-terminal CBD detects and attaches to various epitopes in the cell wall for proper binding of the EAD’s catalytic effects (Matamp and Bhat, 2019). The bacteriophage-derived endolysins of Gram-negative hosts can be organized in different ways, but the majority of Gram-negative endolysins contain a basic globular EAD domain without a CBD (Briers et al., 2007). The new investigation additionally found Gram-negative endolysins with globular structures, with one or two CBDs at the N-terminus and the EAD module at the end of the C-terminal region (Briers et al., 2007).

### 1.3. Classification and characterization of endolysin

Endolysins are classified according to their location of cleavage position. The categories of these enzymes are lysozymes (N-acetylmuramidases), glycosidases (N-acetyl--d-glucosamidases), N-acetylmuramoyl-l-alanine amidases, and L-alanoyl-d-glutamate endopeptidases (Fenton et al., 2010; Abdelrahman et al., 2021). Phage-encoded cell wall hydrolases are a general word that could be used to describe these enzymes. Generally, endolysins consist of one of these four N-terminals linked to a different cell wall-binding domain (Murray et al., 2021).

#### 1.3.1. Glycosidases

Glycosidases break the −1,4 glycosidic linkages that connect the peptidoglycan layer’s changing polymeric structures of N-acetylmuramic acids (MurNAc) and N-acetylglucosamines (GlcNAc). N-acetyl--D-muramidases, which split bonds within MurNAc and GlcNAc; N-acetyl--D-glucosidases, which split bonds within GlcNAc and MurNAc residues (Matamp and Bhat, 2019). Transglycosylases, like the other two glycosidases, cleave −1,4 bonds within MurNAc and GlcNAc, but they are additionally involved in an intramolecular process that leads to the formation of a 1,6-anhydro ring at the MurNAc residue (Rodríguez-Rubio et al., 2016). Bacteria are killed by lysozymes (N-acetylmuramidases) through specific hydrolysis. N-acetyl-β-d-glucosamidases, also known as glycosidases, regulate the hydrolysis of the glycosidic bond. The NAG (N-acetylglucosamine) and NAM (N-acetylmuramic acid) monomers of peptidoglycan polymers are linked together by β-1,4 glycosidic bonds. By hydrolyzing these linkages, lysozyme acts to disrupt the peptidoglycan cell wall’s structural integrity. This causes the turgor pressure to become unbalanced, which leads to the lysis of the bacterial cell (Ragland and Criss, 2017).

#### 1.3.2. Amidases

Peptidoglycan amidases, also known as N-acetylmuramoyl-L-alanine amidases, act by breaking the amide bond that splits the glycan strand from the stem peptide situated between the L-alanine and N-acetylmuramic acid residues (Abdelrahman et al., 2021).

#### 1.3.3. Endopeptidases

Endopeptidases are enzymes that break the bonds between two amino acids in the stem peptide. The peptide made up of the L-alanoyl-d-glutamate link is the target of interpeptide bridge-specific endopeptidases and L-alanoyl-d-glutamate endopeptidases. Bond cleavage can occur within an interpeptide bridge or between stem peptides (Rodríguez-Rubio et al., 2016).

## 2. The significant features of endolysin

### 2.1. Broad lytic spectrum

Endolysin is mainly active in Gram-positive bacteria that lack a protective outer cell membrane. The existence of an outer membrane in Gram-negative bacteria limits but does not entirely prevent the function of endolysin (Murray et al., 2021). Endolysin often has a broader range of specificity than phages, and their specificity is typically at the genus or species level. Endolysins’ selectivity for bacterial targets is due to their cell wall-binding domains, which detect and bind receptors independently to a specific substrate within the target cell wall. Due to this reason, Gram-negative lysins have a wider range of targets, while Gram-positive targeted lysins often have a narrower host range (Ajuebor et al., 2016). Endolysins with broad-spectrum activity against Gram-positive bacteria have been reported. A *pneumococcal* bacteriophage-derived lytic endolysin Pal can eradicate 15 prevalent pneumococcal serotypes, even those with a high level of penicillin resistance (Loeffler et al., 2001). The endolysin phi11 shows potent antimicrobial activities against various *Staphylococcus* strains including *S. aurues, S. epidermidis, S. hyicus, S. simulans, S. xylosus* and *S. wraneri* (Donovan et al., 2006). In many other studies, the endolysins’ efficacy against Gram-positive bacteria has been reported including ClyR, ClyJ, ClyV, ClyC, SAL200, LysK, and Cpl-1 (Yang et al., 2015a; Seijsing et al., 2018; Bae et al., 2019; Gondil et al., 2020a; Huang et al., 2020; Yang H. et al., 2020; Li X. et al., 2021). Some of the examples of endolysin showed broad-spectrum activity against Gram-negative bacteria. The endolysins LysAB54, LysP53 and LysPA26 showed antibacterial activity against multidrug-resistant Gram-negative bacteria such as *A. baumannii*, *Pseudomonas aeruginosa*, *Klebsiella pneumoniae*, and *Escherichia coli* (Guo et al., 2017; Khan et al., 2021; Li C. et al., 2021). The reported endolysin Abtn-4 and LysSS has a wide range of bactericidal activity against a wide range of Gram-positive and Gram-negative bacteria, including *E. coli*, *A. abauminii*, *P. aeruginosa*, *K. pneumoniae*, *Salmonella* spp. and *S. aureus* (Kim et al., 2020; Yuan et al., 2021).

### 2.2. Less resistance

Endolysins are regarded as persistent therapeutic agents in terms of bacterial resistance development. Their specificity for a bacterial species or genus gives them several advantages over broad-spectrum antibiotics. The co-evolution of phages with their host bacterium has brought numerous benefits, including conserved binding ability and activity, as well as a rare possibility of mutation in binding sites, making resistance to endolysins a remarkable finding. Because the majority of antimicrobial resistance mechanisms are within the cell, the extracellular application of endolysins and the presence of target peptidoglycan on the cell’s outer surface prevent the development of resistance to endolysin (Gondil et al., 2020b). The repeated exposure of *Streptococcus pneumoniae* to endolysin Pal at a low concentration shows no resistance (Loeffler et al., 2001). Experiments were performed and attempted to bring resistance in *Bacillus* spp. against endolysin PlyG and methicillin-resistant *S. aureus* against endolysin ClyS. Still, no resistance occurred in both bacteria against their corresponding endolysins, while antibiotic resistance occurred in these bacterial strains using the same methodology (Schuch et al., 2002; Pastagia et al., 2011). There is no published report on the resistance of bacteria to endolysin.

### 2.3. No harmful impact on the normal microbiota

To protect its host from attacking pathogens, the normal microbiota develop a physical barrier (Cash et al., 2006). Any disruption in the natural microbiome can lead to significant disease (Wang et al., 2017). Antibiotics facilitate human health and life span; yet, broad-spectrum antibiotics disturb the existing microbiota, resulting in dysbiosis and adverse effects (Keeney et al., 2014). Endolysins, compared to typical broad-spectrum antibiotics, can target selected bacterial species with minimal adverse effects on the surrounding microbiome, and the absence of bacterial resistance mechanisms has also been reported as a benefit of the use of endolysin. Because of these characteristics, endolysins have been allocated to a new class of antimicrobial agents known as enzybiotics (Murray et al., 2021). PlyV12, a bacteriophage-derived endolysin showed a strong lytic activity against the vancomycin-resistant strains of *Enterococcus faecalis* and *E. faecium*. Endolysin causes less disruption of the normal microbiota because they do not transfer resistance genes or bacterial toxins that harm the colonizing bacteria of mucous membranes (Rahimzadeh et al., 2018). They can stimulate the immune system without neutralizing or inhibiting antibacterial action. As a result, they can be used to treat systemic infections (Fischetti, 2008). Imanishi et al. (2019) investigated the therapeutic potential of a recombinant S25-3 against *S. aureus*. They found out, firstly, that the recombinant endolysin S25-3 was effective against *S. aureus*. Secondly, in an experimental mouse model of impetigo, topical administration of recombinant S25-3 endolysin reduced the number of intraepidermal *Staphylococci*. Thirdly, in the same mice, treatment with recombinant S25-3 endolysin boosted the diversity of the skin microbiota. The Pal *in-vivo* analysis showed that microbial species commonly present in the human oropharynx were not affected by the Pal endolysin (Loeffler et al., 2001). These findings showed that endolysin has no adverse effect on the human microbiota.

### 2.4. Safety

Bacteriophages are part of the human microbiome. Hence bacteriophage-derived endolysin is unlikely to harm human health (Navarro and Muniesa, 2017; Kirsch et al., 2021). Endolysins can surpass the immune system due to their fast action and specific receptors of the CBD on its target host (Jado et al., 2003). Still, their eventual acceptance as antimicrobial agents for the general public heavily depends on their immunogenicity, how the body’s immune system will respond to them, and their safety assessment on human health (Abdelrahman et al., 2021). In this regard, several studies show endolysins’ safety assessment. The safety and toxicity of two *pneumococcal* endolysins, Pal and Cpl-1 were investigated in human macrophages and pharyngeal cells. Similarly, no physical or behavioral changes were observed in mice injected with 15 mg/kg of each endolysin. IgG levels in mice exposed to the *Streptococcus pneumoniae* endolysins Cpl-1 and Pal increased. However, IgE levels remained low, suggesting a low probability of hypersensitivity or allergic reactions. Additionally, no adverse health consequences in mice with increased levels of pro-inflammatory cytokines or complement activation were seen, suggesting that these endolysins had good safety and toxicity profiles. The Pal and Cpl-1’s endolysin safety and toxicity characteristics justify the safe use of endolysin (Harhala et al., 2018). PlyCD1-174 is effective against *Clostridium difficile* spores infecting the large intestine in the presence of its intestinal contents. The PlyCD1-174 was injected into the colons of *C. difficile*-infected mice and 400 μg of endolysin delivered via the colon had no abnormal adverse effects, such as diarrhea or mice weight loss (Wang et al., 2015). In rodent single-and repeated-dose toxicity trials, intravenously administered SAL200 revealed no adverse effect. There were no adverse findings in the dog repeated-dose toxicity test, except temporary abnormal clinical symptoms found in certain dogs when daily injection of SAL200 was continued for more than one week. All aberrant findings found in these safety review investigations were minor and were rapidly recovered and no animal deaths were reported in the safety assessments (Jun et al., 2014). In a clinical trial, healthy male volunteers were administered the endolysin SAL200, and the results showed good endolysin tolerance in human subjects. Therefore, endolysins have an excellent safety profile and revealed no side effects like fever, stomach cramps, or diarrhea in preclinical studies conducted using animal models, demonstrating their safety aim (Jun et al., 2017). The safety and immunogenicity efficacy of endolysin ClyR was investigated in a systemic *Streptococcus agalactiae* infection mouse model. The ClyR rescued the mice and had no harmful effects on the mice (Yang et al., 2015a). The endolysin MV-L safety activity is evaluated in the mice. The mice who received a single high dosage of endolysin followed by a repeat dose did not show any side effects or a change in their survival rate (Rashel et al., 2007). The endolysin PlyC, immunogenicity, and allergic reactions were tested in mice and human serum samples from healthy volunteers. Mice were challenged with PlyC, and humans lacked PlyC-specific IgE. PlyC was immunogenic but did not cause hypersensitivity. Furthermore, adverse effects, particularly those indicating hypersensitivity, were closely monitored, and no adverse reactions were recorded. These findings significantly support PlyC’s safety as a possible therapeutic enzyme that can be applied to treat severe bacterial infections (Harhala et al., 2022). The endolysin LysGH15 is tested for the influence of endolysin inactivation by antibodies. LysGH15-specific antibodies did not impact LysGH15’s ability to kill MRSA *in vitro* or *in vivo* (Zhang et al., 2016). The high safety of endolysins could be because endolysin is very specific for distinctive and conserved structures of bacterial cell wall peptidoglycan or polysaccharides that are not present in mammalian cells (Nau and Eiffert, 2002; Nelson et al., 2012). Although the findings of safety studies to date support the claim of endolysins as a potential safe antibacterial agent. More research in this area is needed to confirm that different classes of endolysin do not pose a significant concern to the host (Murray et al., 2021).

## 3. The applications of endolysins for food safety

### 3.1. The applications of endolysin against bacterial biofilm

Biofilms are microorganism communities that are sessile and embedded in a self-produced extracellular environment, being the most widespread bacterial lifestyle (Qian et al., 2022). The well-organized structure of these multilayer colonies guards bacterial cells against external threats, improving their ability to tolerate antibiotic or disinfectant treatment. These colonies are identified by enhanced antibiotic resistance in both hospitals and foods. They are a persistent cause of re-infection and re-contamination of foods (Abee et al., 2011; Fernández et al., 2018). Bacterial biofilm formation is a defense mechanism that bacteria use to deal with unfavorable conditions such as food scarcity or toxic antimicrobial dosage. Antimicrobial drugs often have a limited effect on biofilms, which is hypothesized to be owing to several biofilm-specific features. Low growth rates hamper antibiotics, and evidence showed that sessile bacteria in biofilms have a different metabolic state. Antibiotics’ diffusion velocity is also restricted within biofilms. Reduced antibiotic penetration increases the antibiotic concentration in the biofilm’s deeper layers, allowing biofilm cells to adapt to the antibiotic through stress-induced metabolic and transcriptional changes (Srinivasan et al., 2021). Because of their considerable resistance, the *S. aureus* biofilms are recognized as a serious concern in the food industry. The reported endolysins LysCSA13, ϕ11, Lys109, LysP108, XZ.700 and LysK did the eradication of bacterial cells of the *S. aureus* and eliminated its biofilms from different surfaces, including polystyrene, glass, and stainless steel. These endolysins can be used as a potential biocontrol agent in the different food processing sectors (Sass and Bierbaum, 2007; Schmelcher et al., 2015; Cha et al., 2019; Kuiper et al., 2021; Lu et al., 2021; Son et al., 2021).

The *L. monocytogenes* and *Salmonella* are also increasingly pressing issues for food industries because of their biofilm formation. The recombinant endolysins PlyLM and 293-amidase showed anti-biofilm activity against *L. monocytogenes* while the endolysin Lys68 showed a synergistic biofilm-reducing activity against the *salmonella* in combination with malic or citric acid. Lys68 has good thermo-stability properties so it can be an alternative antibacterial protein against Gram-negative pathogens in the food industry (Simmons et al., 2012; Oliveira et al., 2014; Pennone et al., 2019).

### 3.2. Endolysin applications for veterinary

Antibiotic misuse and overuse have resulted in a significant emergence of resistant bacteria in meat-producing animals, particularly cattle, poultry, and pigs, which have been identified as crucial carriers of antibiotic-resistant bacteria and their particular genes, which can transfer to humans through direct contact or indirectly through foods (Marshall and Levy, 2011). In response to increasing concern over antibiotic misuse in food-producing animals, many large food companies have agreed to eliminate antibiotic use and improve control over alternative treatments. This creates complex scenarios for the animal-husbandry sector, which can be overcome using bacteriophage-derived endolysins (Love et al., 2018). The poultry market has faced severe problems due to *Clostridium perfringens*, which can infect 95% of poultry (Timbermont et al., 2011). The endolysin phiSM101 showed a wide range of bactericidal activity against *C. perfringens* (Tamai et al., 2014). *Salmonella* can cause widespread disease in chickens, and it causes economic losses in the poultry business (Wernicki et al., 2017). The endolysin LysSE24 derived from the *Salmonella* bacteriophage LPSE1, showed antibacterial activity against the *Salmonella* strains (Ding et al., 2020).

### 3.3. Endolysin applications for meat

Meat is a significant carrier of foodborne infections (Bantawa et al., 2018). The endolysin LysSA11 showed a 3-log CFU/mL of methicillin-resistant *S. aureus* (MRSA) bacterial reduction in ham meat at a refrigerator temperature of 4°C (Chang et al., 2017a). The endolysin Trx-SA1 effectively reduce *S. aureus* in the cow udders. Trx-SA1 can be used as an alternate bio-preservative to minimize *S. aureus* contamination during food storage (Fan et al., 2016).

### 3.4. Endolysins applications for dairy products

Dairy-borne pathogens, such as *L. monocytogenes, S. aureus, Salmonella,* and Shiga toxin-producing *E. coli* (STEC), should be controlled in the dairy production industry. The lysis activities of endolysins have been investigated particularly in dairy products (Lee et al., 2022). *S. aureus* has been effectively eliminated by several endolysins in milk (Obeso et al., 2008; García et al., 2010; Chang et al., 2017a). The endolysins LysH5, LysSA97 and LysSA11 were tested in milk which showed strong bactericidal activity against *S. aureus*. LysH5 revealed a synergistic bactericidal activity in pasteurized milk when used in combination with nisin. These antimicrobial proteins can be effective milk preservative candidates (Obeso et al., 2008; García et al., 2010; Chang et al., 2017a,b). The *C. tyrobutyricum* has been linked to the late blowing of hard and semihard cheese. Even minute quantities of heat-resistant spores can result in food spoilage during pasteurization, processing, and canning procedures. The *C. tyrobutyricum* phage ϕCTP1 endolysin Ctp1L is an antibacterial protein that showed activity in milk and prevents late blowing during cheese manufacturing (Mayer et al., 2010). *Listeria* contamination must be eliminated when making fresh cheese since its delicate taste and textures are inconsistent with several antimicrobial treatments and chemicals commonly used in other foodstuffs. The endolysin PlyP100 can potentially prevent the spread of *L. monocytogenes* in fresh cheese (Van Tassell et al., 2017). The endolysins LysZ5, ply511, and ply118 lysis the *L. monocytogenes* in milk. LysZ5 has good host specificity and host lysis activity at refrigerator conditions. ply511 and ply118 showed broad-spectrum activity and had high thermal resistance (Zhang et al., 2012; Schmelcher et al., 2012c). The endolysin ply100 showed synergistic bactericidal activity in combination with nisin against the *Listeria* in the Queso Fresco cheese (Ibarra-Sánchez et al., 2018). Endolysins are also tested against foodborne *Listeria* in milk, soy milk and Queso Fresco cheese (Zhang et al., 2012; Schmelcher et al., 2012c; Ibarra-Sánchez et al., 2018).

### 3.5. Endolysins applications in vegetables

To reduce and prevent the antibiotic resistance pathogenic bacteria in vegetables, endolysin has also been tested in vegetable products. Several endolysins have been proposed as possible antibacterial bio-preservative agents for vegetable products against *L. monocytogenes* in iceberg lettuce (Schmelcher et al., 2012a). The endolysin LysP53 can lysis *Salmonella enteritidis* on fresh romaine lettuce and can be employed as a biocontrol agent to lower bacterial burdens in fresh vegetable food due to its good activities against a wide range of bacteria, thermal stability, and safety (Li et al., 2023). The endolysin cpp-lys greatly lysis the bacteria *C. perfringens* which produced various toxins. The activity is checked and confirmed on lettuce. The results of this investigation showed that endolysin cpp-lys has potential uses in the food industry for the control of *C. perfringens* (Zhao et al., 2023). LysWL59 and LysWL60, are potential bacteriophage LPST10-derived endolysins, which revealed extensive lytic activity and diminished viable *S. typhimurium* on lettuce (Liu et al., 2019).

### 3.6. Endolysins and its CBD for fast detection of pathogenic bacteria in food

To ensure food safety, sensitive and specific diagnostic protocols that accurately and rapidly detect pathogenic bacteria in food are essential (Hagens and Loessner, 2007). A useful tool for the detection of a particular food-borne bacteria is CBD which has been fluorescently tagged (Loessner et al., 2002). CBDs derived from bacteriophage-derived endolysins are excellent choices for making use of novel detection tools due to their high degree of specificity and binding affinity. CBDs from *Listeria* phage endolysins are a prime example, as their binding affinity to the target cells closely resembles or exceeds antigen–antibody interactions (Loessner et al., 2002; Schmelcher et al., 2010). Furthermore, CBDs have highly specific yet varied binding spectra, ranging from the level of the genus to the level of the serovar or even the level of the strain, providing new opportunities for the detection, immobilization, and differentiation of cells (Schmelcher et al., 2010). The endolysin LysCPAS15 inhibited the bacterial cells by up to a 3-log reduction, and enhanced green fluorescent protein EGFP-fused CBD protein (EGFP-LysCPAS15_CBD1) detects *C. perfringens* (Cho et al., 2021). A novel method for detecting *S. aureus* in milk is developed by combining immunomagnetic separation with the enzyme-linked CBD of endolysin plyV12, which binds *S. aureus* with high affinity, tested in the contaminated milk. On the cell surface of *S. aureus*, there are millions of binding sites for CBD attachment, which significantly improves the detection sensitivity. This method could be used to detect *S. aureus* in different food samples due to its simplicity, fast detection, and high sensitivity (Yu et al., 2016). Fused CBDs were developed to target and detect other bacterial pathogens, such as *B. cereus and L. monocytogenes* (Ahmed et al., 2007; Eugster et al., 2011; Kong and Ryu, 2015). However, due to the outer membrane barrier, Gram-negative food-borne pathogens cannot be detected by CBD, which is a significant drawback. CBD cocktails can recognize numerous food-borne bacterial pathogens that are present in a food sample at once by using various colored fluorescent tags. For instance, a multiplex decorating with CBDs allowed the identification of various serovar groups of Listeria. The CBD-P35 and CBD500 were able to recognize various *Listeria* strains in both milk and camembert cheese samples when they were labeled with different fluorescent markers including RedStar and GFP (green fluorescent protein) (Schmelcher et al., 2010). Due to high specificity and binding activity for pathogenic bacteria, CBD is a perfect option to replace antibodies in the rapid detection of bacterial pathogens (Bai et al., 2016). Even though there has been tremendous advancement in the use of endolysins or their CBDs for detecting pathogenic bacteria in food, future studies should focus on further investigation of endolysins or their CBDs into the quickly developing biosensor technology (Schmelcher and Loessner, 2016).

## 4. The use of endolysins for the prevention of foodborne bacterial pathogens

### 4.1. Salmonella

**Figure 2:**
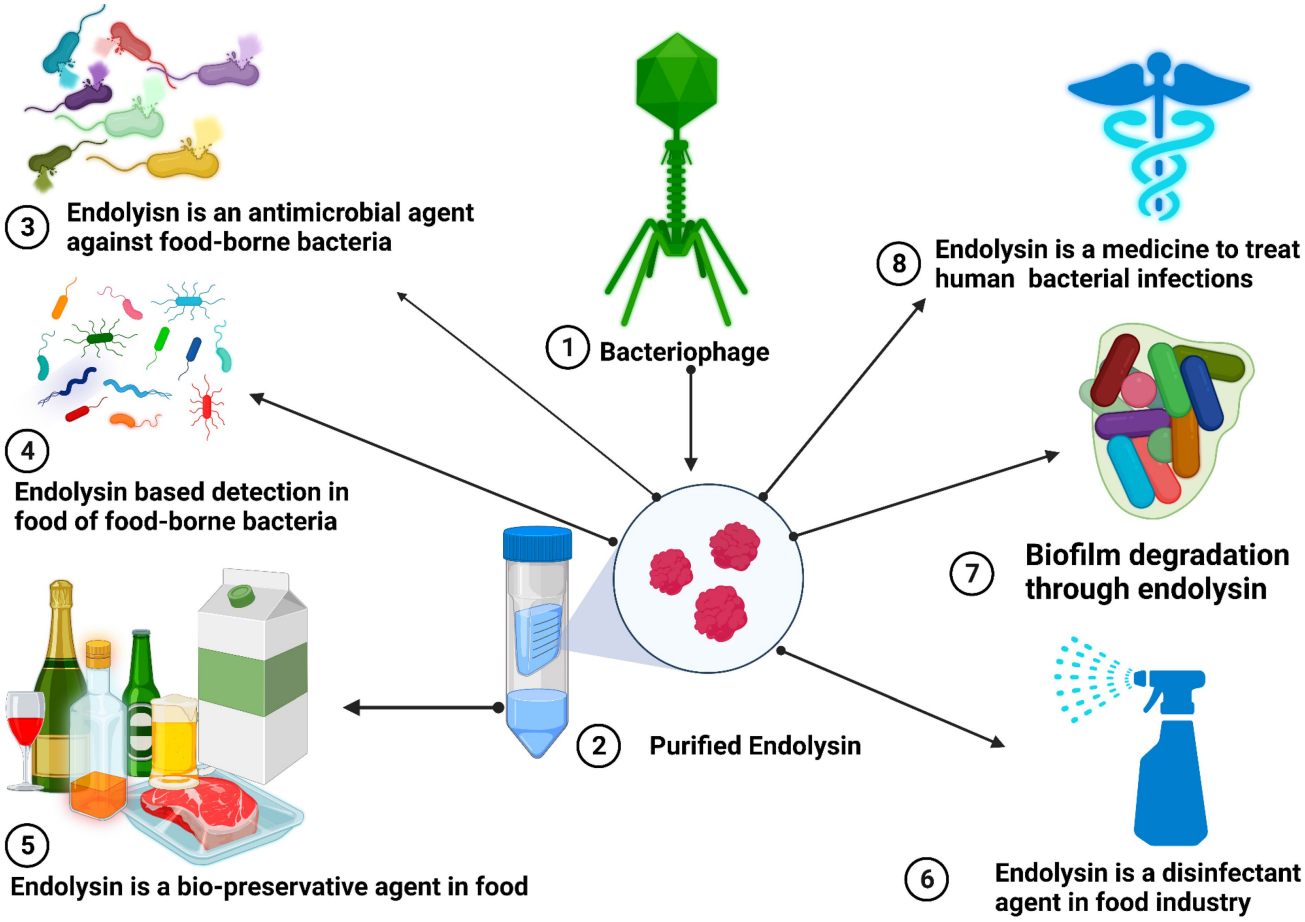
Antimicrobial properties of bacteriophage-derived endolysins for the safety of humans and foods from pathogenic bacteria. Endolysin is a bio-preservative agent in food chains and biofilm degradations through endolysin. Endolysin-based detection in food of food-borne bacteria, endolysin as a disinfectant agent in the food industry, and its use in the clinic as a therapeutic agent to treat and cure human antibiotic-resistant bacterial pathogens.

*Salmonella* is usually recognized as one of the most significant and widespread foodborne pathogens in the entire world. *Salmonella* has over 2,000 serotypes, some of which can cause human enteric infections, also known as salmonellosis (Majowicz et al., 2010). *Salmonella* nontyphoidal, which causes food poisoning outbreaks, is China’s second most common foodborne pathogen (Fu et al., 2019). LysSTG2, En4 and SPN9CC bacteriophage-derived endolysins showed bactericidal activity against *S. typhimurium*. The LysSTG2 (100 μg/mL) and slightly acidic hypochlorous water (40 mg/L of chlorine) eliminated more than 99% of the *S. typhimurium*. En4 showed a reduction of *S. typhimurium* 1.0–1.6 log CFU/g in frozen and thawed raw chicken meat with the addition of 0.1% NaHCO3. These results suggested that using endolysin together with permibilizers could be a novel and effective way to combat *S. typhimurium* in the food industry (Lim et al., 2014; Zhang et al., 2021; Abhisingha et al., 2023). Figure 2 describes the different applications of endolysin.

### 4.2. *Escherichia coli* O157: H7

*Escherichia coli* O157: H7 is a foodborne pathogen that causes a wide range of health issues in humans, including lethargy, nausea, bloody diarrhea, and, in extreme cases, death (Alhadlaq et al., 2023). The fresh vegetables are an essential part of a healthy diet. However, the contamination of salad greens with *Escherichia coli* O157: H7 has resulted in serious illness and significant economic losses (Xu et al., 2021). The endolysins PlyEc2 and RLysJN01 showed bactericidal activities against the *E. coli* O157:H7 on lettuce and did not affect the color or taste quality of the lettuce (Xu et al., 2021; Shen et al., 2022).

### 4.3. Staphylococcus aureus

*Staphylococcus aureus* is the most common cause of foodborne illness around the globe (Kadariya et al., 2014). MRSA has been a considerable concern to public health worldwide. During the previous decade, an extensive spread of MRSA with cattle origin has been detected along the farm-to-fork food chain (Köck et al., 2014). One potential control technique is purified endolysins to serve as bio-preservatives in food products to control *S. aureus* food poisoning. The endolysins Lys109 inhibited *S. aureus* and MRSA in milk and bacon foods and has promising lysis activity on planktonic bacteria and biofilms (Son et al., 2021). CHAPSH3b (which includes the endopeptidase domain of HydH5 and the cell wall targeting domain of lysostaphin) has been reported which eliminate *S. aureus* in milk to undetectable levels and retained significant activity in raw milk, after pasteurization, and during storage at 4°C. For these reasons, CHAPSH3b has been proposed as a possible bio-preservative component for milk storage (Rodríguez-Rubio et al., 2013).

### 4.4. Listeria monocytogenes

*Listeria monocytogenes* is a bacterium found in ready-to-eat foods such as meats, mixed salads, dairy, and vegetables (Zhu et al., 2017; Desai et al., 2019). *L. monocytogenes* is an infectious pathogen that can transfer to humans through food contamination. It can cause severe infections with possibly deadly outcomes in vulnerable groups like newborns, the old, pregnant women, and immunocompromised people (Vázquez-Boland et al., 2001; de Noordhout et al., 2014). Recently, researchers have studied, examined, and reported endolysin as a natural biocontrol agent against the foodborne pathogen *L. monocytogenes*. The endolysin PHA_BNPs showed prominent activity against *L. monocytogenes* (Stone et al., 2022). Fresh Hispanic cheese, known as queso fresco, is frequently linked to outbreaks of listeriosis in the US and human foodborne diseases caused by *L. monocytogenes*. The endolysins PlyP40 and PlyPSA prevented and eradicated *L. monocytogenes* contamination in queso fresco at cold storage for 28 days. These endolysins showed activity at low temperatures, pH, and salinity (Holle and Miller, 2021). The heat-stable *L. monocytogenes* endolysins, PlyP35, Ply511, and Ply118, is a fascinating finding, which shows lysis activity after 30 min of heating to 90°C. Ply511 and Ply118 decreased the amount of live *L. monocytogenes* when added to artificially spiked food items including iceberg lettuce and whole cow milk (Schmelcher et al., 2012a). These stable heat endolysin can be used in heat-treated food products like pasteurized milk (Schmelcher et al., 2012c). The endolysin LysZ5 from the *L. monocytogenes* phage FWLLm3 specifically reduced host growth up to 4 log CFU in soya milk in 3 h at 4°C, indicating that LysZ5 has excellent host specificity and host lysis activity at the refrigerator temperature (Zhang et al., 2012). Ply500, a listericidal peptidase, revealed a broad activity range across the genus *Listeria* (Schmelcher et al., 2012c).

### 4.5. Clostridium

*Clostridium perfringens* is a Gram-positive, anaerobic, and rod-shaped bacteria that can cause food poisoning through its enterotoxin (Baldassi, 2005). Fast detection and treatment of *C. perfringens* infection are essential for maintaining food safety (Cho et al., 2021). It has been reported that *C. perfringens* frequently contaminates milk (Mcauley et al., 2014). The endolysin LysCPAS15, Psa, Psm, PlyCP10 and PlyCP41 showed lytic activity against *C. perfringens* and as natural antibacterial proteins for preventing the spread and contamination in milk, meat and other food products. LysCPAS15 was tested in milk which reduced the bacterial load in milk. The PlyCP10 and PlyCP41 inhibited the growth of seventy-five *C. perfringens* strains isolated from pigs, chickens, and cows (Swift et al., 2018; Cho et al., 2021; Sekiya et al., 2021). *Clostridium botulinum* is a Gram-positive, anaerobic bacterium that produces spore’s botulinum neurotoxin (BoNT). BoNT causes botulism, a potentially fatal flaccid paralysis, in humans and animals. The germination and development of *C. botulinum* spores in food can cause different infections in humans and animals (Zhang et al., 2020). The endolysin CBO1751 efficiently lyses *C. botulinum* and also eliminates neurotoxin production. The antibacterial endolysin CBO1751 against *C. botulinum*, is a novel anti-botulinum protein for non-thermal applications in food and agriculture (Zhang et al., 2020). *Clostridium sporogenes* are spoilage bacteria that can cause food spoilage and rotting problems (Mayer et al., 2012). The endolysin CS74L showed lysis activity against the 16 tested strains of *C. sporogenes* (Mayer et al., 2012).

### 4.6. Vibrio parahaemolyticus

The main pathogen involved in seafood-borne bacterial infections is *Vibrio parahaemolyticus*. *V. parahaemolyticus* is a gram-negative bacterium inhabiting sea foods, including fish, shrimp and oysters (Letchumanan et al., 2014). The *V. parahaemolyticus* forms biofilms commonly in food manufacturing environments, contaminating food manufacturing machinery and products (Lopatek et al., 2018). *V. parahaemolyticus* biofilm production can increase resistance to antibacterial drugs commonly used in food, raising the chance of pathogen infections (Ning et al., 2021). Food contaminated with *V. parahaemolyticus* can cause stomach pain, nausea and vomiting, acute dysentery, and bloodstream infections (Baker-Austin et al., 2017). Additionally, due to antibiotic abuse, *V. parahaemolyticus* antibiotic resistance is becoming a severe concern to consumers (Shaw et al., 2014). Ninety *V. parahaemolyticus* strains from the main seafood in the China provinces of Shandong, Hebei, and Liaoning near the Bohai and Yellow Seas were recently examined for antibiotic resistance. Almost half of the isolates had at least three drug resistances, and more than 80% were resistant to ampicillin and cephazolin (Jiang et al., 2019). The demand for alternate antimicrobials to manage *V. parahaemolyticus* and associated biofilms may have recently increased (Ning et al., 2021). Endolysins are prospective substitutes for conventional antibiotics in the fight against *V. parahaemolyticus* and its biofilms (Matamp and Bhat, 2019). The recombinant endolysins Lysqdvp001, LysVPMS1 and LysVPp1 inhibited the growth of *V. parahaemolyticus*. Lysqdvpoo1 and poly-lysine showed robust synergistic antibacterial activity, so these two can be used in combination to tackle *V. parahaemolyticus* and its biofilms in the food sector (Wang W. et al., 2016; Li et al., 2018; Zermeño-Cervantes et al., 2018; Ning et al., 2021).

### 4.7. Campylobacter jejuni

*Campylobacter jejuni* is the most common foodborne bacteria which can cause gastroenteritis worldwide and poses a severe risk to public health and food safety (Moffatt et al., 2016). The endolysin Cj1 and Cj5 can eradicate and control the *C. jejuni* in chicken food models in a refrigerated 5°C (Zampara et al., 2021).

**Figure 3:**
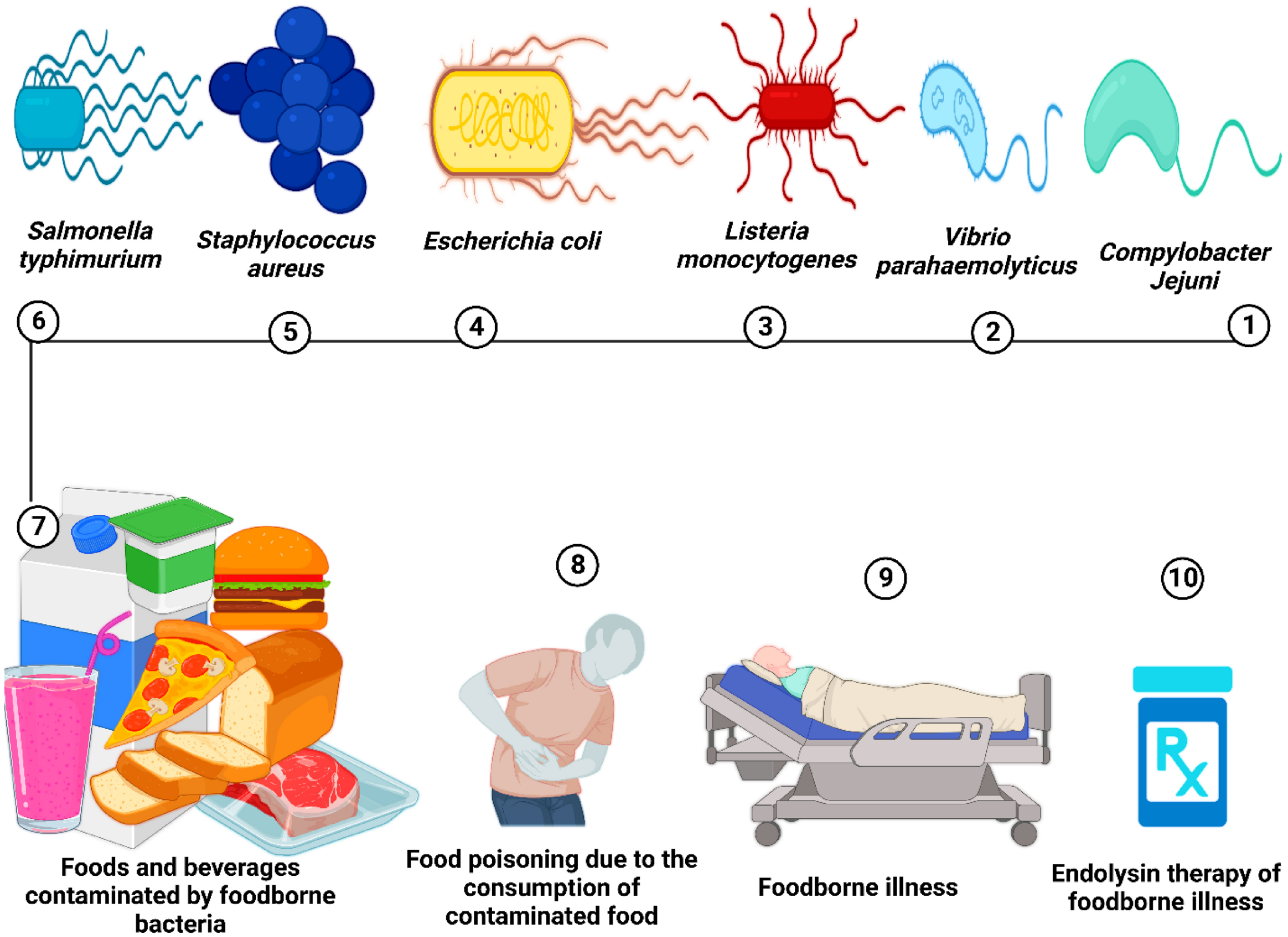
Food is contaminated by food-borne pathogenic bacteria, leading to food poisoning and potentially severe food-borne illnesses. Endolysin therapy could serve as one of the effective treatments for food-borne bacterial infections.

Figure 3 defines the efficacy of endolysin as an antibacterial agent against foodborne illness caused by foodborne bacterial pathogens. Table 1 summarizes the use of endolysins in different food products, while Table 2 describes the efficacy of endolysin against different pathogenic bacteria.

**Table 1:** The use of endolysins in different food products.

Antibacterial activity spectrum against the target host	Lysin	Main results	Applications in Food	Reference
*S. aureus*	Lysdb	Break the 6-O-acetylated peptidoglycans existing in *S. aureus* cell walls.	Cheese	Guo et al. (2016)
*S. aureus*	LysSA97	Synergistic bactericidal activity with carvacrol (oil) in skim milk and whole milk.	Milk	Chang et al. (2017b)
*S. aureus*	LysSA11	Within the 15 min at 4°C, 3-log CFU/mL bacteria cells lysis in meat that had been experimentally contaminated with MRSA. Decontaminant in steel knives and plastic chopping.	Ham meat	Chang et al. (2017a)
*S. aureus*	LysH5	8 logs CFU/ml, Bacterial cells reduction in milk. Broad host range against clinical *staphylococcus*, including *S. epidermidis* and *S. aureus*. Synergistic sterilizing activity against *S. aureus* in combination with nisin in dairy.	Milk	Obeso et al. (2008)
*S. aureus*	HydH5Lyso, HydH5SH3b, CHAPSH3b	HydH5 and CHAPSH3b revealed significant bactericidal action in skim and whole milk at 37°C and 25°C.	Whole and skim milk.	Rodríguez-Rubio et al. (2013)
*S. aureus*	PlyV12	High specificity of the detection of *S. aureus* in milk.	Spiked milk	Yu et al. (2016)
*S. aureus* and *Bacillus cereus*	LysB4EAD-LysSA11	Good cell lysis activity against *S. aureus* and *B. cereus*. Within only 2 h, therapy with 3.0 μM LysB4EAD-LysSA11 reduced *S. aureus* and *B. cereus* in rice to zero levels.	Boiled rice	Son et al. (2020)
*L. monocytogenes*	PlyP100	Sustainable in Queso Fresco for 28 days. High retention rate in cheese curd. High stability during cold storage. Dose-dependent listeriacidal.	Queso Fresco (cheese)	Ibarra-Sánchez et al. (2018)
*L. monocytogenes*	Ply511, Ply118	Wide spectrum activity against *Listeria* strains. High heat resistance.	Cow milk	Schmelcher et al. (2012c)
*L. monocytogenes*	PlyP825	Synergistic antibacterial activity with hydrostatic pressure.	Milk	Misiou et al. (2018)
*L. monocytogenes*	PlyP40 and PlyPSA	PlyP40 is effective against a wide variety of *Listeria*. PlyP40 and PlyPSA effectively reduce *L. monocytogenes* numbers in queso fresco. PlyP40 and PlyPSA can keep their lytic activity under refrigerated conditions.	Queso fresco	Holle and Miller (2021)
*L. monocytogenes*	LysZ5	4-log CFU/mL antibacterial reduction in soya milk at four °C within three hours.	Soya milk	Zhang et al. (2012)
*Escherichia coli O157:H7*	PlyEc2	Effective decontamination agent. In the Romaine lettuce decontamination model, eliminating 99.7% of *E. coli* O157:H7 cells. The taste and texture of the treated leaves are unaffected.	Romaine lettuce	Xu et al. (2021)
*Salmonella Typhimurium*	LysWL59 and LysWL60	LysWL59 and LysWL60 showed a wide range of catalytic action toward Gram-negative bacteria exposed to chloroform. Treated with 2.5 mM LysWL59 and 0.5 mM EDTA, *S. typhimurium* cells on lettuce were reduced by 93% in 1 h.	lettuce/ Salad (Vegetables)	Liu et al. (2019)
*Clostridium perfringens*	LysCPAS15	Bacterial cells were reduced in milk by up to 3 logs in two hours. Rapid and specific detection of *C. perfringens* in milk.	Milk	Cho et al. (2021)
*Clostridium tyrobutyricum*	Ctp1l	Moderate bactericidal activity of the host bacterium	Cow Milk	Mayer et al. (2010)

**Table 2:** The applications of endolysin against different pathogenic bacteria.

Lysin	Antibacterial activity spectrum	Significant features	Reference
PlyC	*Streptococcus* spp.	A powerful antibacterial endolysin was tested in a human and a mouse model. PlyC showed no undesirable symptoms and no hypersensitivity reaction. Potentially safe therapeutic endolysin used to treat bacterial infections.	Harhala et al. (2022)
LysAB54	*Escherichia coli* *Acinetobacter. baumannii* *Pseudomonas aeruginosa* *Klebsiella pneumoniae*	Natural novel Lysin. Rapid bactericidal activity. 100 μg/mL at 37°C for 10 min incubation can make a 4-log reduction. A broad spectrum of antimicrobial activity. Active against all the 40 tested gram-negative bacterial strains.	Khan et al. (2021b)
LysP53	*E. coli* *A. baumannii* *P. aeruginosa* *K. pneumoniae*	Engineered Chimeric Lysin. After 1 h of treatment to 100 μg/mL, bacteria were reduced by 5 logs. Higher decolonization efficacy in the mouse model of burn infection. A broad spectrum of antimicrobial activity.	Li C. et al. (2021)
LysPA26	*E. coli* *K. pneumonia* *A. baumannii* *P. aeruginosa*	Natural Phage derived Lysin. 50 μg/mL can make 4 log reduction in 30 min. Eliminate biofilm formation. Retained good thermostability.	Guo et al. (2017)
PlyE146	*E. coli* *A. baumannii* *P. aeruginosa*	At a 400 μg /ml dose, *E. coli* and *P. aeruginosa* (3 to 3.8 log10 CFU/ml) were prevented after 2 h of treatment at 37°C. *Acinetobacter baumannii* (4.9 to 5 log10 CFU/mL) reduction.	Larpin et al. (2018)
LysABP-01	*E. coli* *A. baumannii* *P. aeruginosa*	20 μM (500 μg/mL) inhibited bacterial growth. Break down the crude cell wall of *E. coli, A. baumannii, and P. aeruginosa* strains.	Thummeepak et al. (2016)
Ply6A3	*E. coli* *A. baumannii* *P. aeruginosa,* *K. pneumoniae* MRSA	Ply6A3 (2 mg/mL) and lysozyme (2 mg/mL) can degrade the bacterial cells and generate a clear ring using the diffusion method. No side effects were observed after intraperitoneal injection into mice. The antibacterial effect of lysins PD-6A3 and Ply6A3 was better than a cocktail of 14 phages.	Wu et al. (2019)
LysAB2	*A. baumannii* *S. aureus*	Strong antibacterial activities against a broad range of Gram-positive and Gram-negative bacteria. The bacteria’s cells lysis significantly after exposure to 500 μg/ml of LysAB2 for up to 60 min. Lyse the cell walls of *Staphylococcus aureus* and peptidoglycan of *A. baumannii*.	Wu et al. (2019)
EndoT5	*E. coli*	4 to 5 log lysis activity of stationary phase of bacterial culture with the addition of permeabilizing agents. T5 endolysin in use with polymyxin B (0·4 μg m/ml) or chlorhexidine (0·5 μg/mL) decreased the number of CFUs by 5 log reduction and in use with poly-l-lysine (80 μg/mL) by 4 log reduction. Thermostable endolysin; After 30 min of heating at 90°C, it retained 65% of its initial activity.	Shavrina et al. (2016)
Lysep3	*E. coli* *P. aeruginosa*	In combination with EDTA (25 mM), Lysep3 does lysis of *E. coli* and *P. aeruginosa.* vB EcoM-ep3 (Phage of Lysep3) can lyse 9 out of 15 clinical isolates of MDR pathogenic *E. coli* from chickens.	Lv et al. (2015)
Art-175	*Escherichia coli isolates*	Colistin and Art-175 disrupt Gram-negative bacteria’s outer membrane. Art-175 killed all colistin-resistant *E. coli* strains isolated from chickens, cows, and pigs. No cross-resistance between Art-175 and colistin.	Schirmeier et al. (2018)
AcLys	*E. coli* *P. aeruginosa* *A. baumannii* *K. pneumonia*	Antibacterial activity against Gram-negative bacteria, resulting in a 1.52 (CFU)/mL reduction in live bacteria culture. AcLys, a “natural artilyzed” enzyme with a C-terminal-helical domain, has a significant anti-Gram-negative bacterial potential.	Sykilinda et al. (2018)
LysSS	*E. coli* *Salmonella* *A. baumannii* *K. pneumonia* *P. aeruginosa* *MRSA*	Both Gram-positive and Gram-negative bacteria are susceptible to LysSS. Show activity against biofilm. Not cytotoxic to human cells. With an Intraperitoneal injection of LysSS (125 ug/mL), 40 per cent of mice were rescued from *A. baumannii* systemic infection.	Kim et al. (2020)
Lys68	*S. typhimurium* *E. coli* *P. aeruginosa* *P. fluorescens* *A. baumannii*	Broader spectrum lysis efficacy (1.5–3 log decrease). Lytic activity against the bacterial biofilm and its stationary phase. Lys68 showed more good activity against *Pseudomonas* strains. Citric acid with Lys68 and malic acid with Lys68 acid have broad antibacterial effects, especially against *S. typhimurium* LT2, which caused 3 to 5 log reductions. Lys68 shows good thermo-stability properties.	Oliveira et al. (2014)
SPN9CC	*E. coli*	0.5 μg/ml lysed all 23 gram-negative bacterial strains tested within 5 min. A broad spectrum of antimicrobial activity. Log phase bacteria are killed by 2 log reduction. With OMP (100 mM EDTA), its activity is increased by four logs reduction. Active and stable at a broad range of temperatures from 24 degrees to 65°C. It revealed maximum lytic activity at 50°C.	Lim et al. (2014)
SPN1S	*S. typhimurium* *E. coli*	It is classified into two subunits, one for enzymatic activity and the other for peptidoglycan binding activity. It has good lysis activity against bacterial cell peptidoglycans. Bactericidal activity against a wide spectrum of Gram-negative bacteria.	Park et al. (2014)
Ply17	*P. aeruginosa* *E. coli* *S. aureus* *S. epidermidis*	Ply17 in various concentrations was tested. Ply17 at 500 μg/mL decreased the viable numbers of EDTA-treated PAO1 by 1 log unit. 3 log killing activity against the log phase of bacteria. Showed better lytic activity at 37°C and 7.5 pH. Broad-spectrum antimicrobial activity against Gram-positive and Gram-negative species. Ply17 can split the peptidoglycan layer of bacteria.	Yang et al. (2018)
Lys394	*E. coli*	Lawn *E. coli* colony-forming unit’s ability was reduced by four orders of magnitude after 30 min of room temperature treatment with 25 μg of Lys394, 1 mM EDTA, and 50 μg g/ml of PGLa peptide. Lys394 was identified as an endopeptidase by *in silico* amino acid sequence analysis. At pH 8.5 and low ionic strength, enzyme activity peaked. It shows the lysis activity of planktonic bacteria in a lysin dose-dependent manner.	Legotsky et al. (2014)
*Salmonella* phage endolysin Gp110	*Salmonella* *P. aeruginosa*	Antimicrobial property toward\ *P. aeruginosa* PAO1 and *S. typhimurium* LT2. Heat resistance endolysin. At 20 to 90°C temperatures, Gp110 remains active.	Rodríguez-Rubio et al. (2016)
Ap3gp15	*E. coli* *P. aeruginosa* *S. typhimurium* *K. pneumoniae,* *B. cenocepacia*	AP3gp15 is a lysozyme that inactivates the PG-1,4-glycosidic bond, releasing GlcNAc and MurNAc. AP3gp15 is two times more potent than available commercial lysozyme. Active against *S. enterica Typhimurium, E. coli, K. pneumoniae, P. aeruginosa, B. cenocepacia,* and *E. coli.* Heat-sensitive but relatively stable at low temperatures. No cytotoxic effect on mammalian cells. 50 μg /ml of Ap3gp15 had no side effects on cell lines.	Maciejewska et al. (2017)
Ply500	*L. monocytogenes*	Highly active against *Listeria innocua*. A 3-log and close to 5-log reduction was obtained after 24 h using 10 and 20 mg/mL of an enzyme, respectively, for a 10^5^ CFU/mL listeria cell challenge.	Solanki et al. (2013)
λSA2-E-Lyso-SH3b and λSA2-E-LysK-SH3b	*S. aureus*	Intensely active in milk. 100 μg /ml in 3 h, diminish the bacterial load of *S. aureus* in the processed cow milk.	Schmelcher et al. (2012b)
ClyC	*S. aureus*	Highly active chimeric Lysin. Showed 9 Log reduction against *S. aureus*. In a mouse infection model of *S. aureus*, a single intraperitoneal injection dose of 0.1 mg/mouse of ClyC considerably increased survival rates. It reduced the bacterial loads in the infected mice’s organs by 2 Log10 (CFU/mL).	Li X. et al. (2021)
ClyH	*S. aureus*	Active against planktonic MRSA cells. ClyH eradicated MRSA biofilms in a time-dependent manner via cell lytic activity. Viable plate counts and kinetic analyses revealed that biofilms of differing ages were susceptible to ClyH. ClyH 10 μg /ml reduced biofilm biomass against methicillin-sensitive *S. aureus* (MSSA) and MRSA strains.	Yang et al. (2014a)
ClyH	*S. aureus*	ClyH MICs against *S. aureus* strains ranged from 0.05 to 1.61 mg/L. In a mice infection model, one dose of ClyH kept mice safe from the demise of MRSA infection. No side effects. No evidence of side effects.	Yang et al. (2014b)
ClyF	MRSA	ClyF has the best MRSA biofilm disruption activity. Antimicrobial activity against a broad range of *S. aureus* strains. In mice, bacteremia and wound infection models, a single treatment of ClyF showed good MRSA removal activity.	Yang et al. (2017)
SiBP1-ClyF	*S. aureus*	Good bactericidal activity against *S. aureus.* Strong antibacterial and antibiofilm properties. 12.5 μg/mL proteins, triggering a decline of 5.52-log10 in viable bacterial number. Effective in killing MRSA (>99.999 per cent within 1 h). Inhibiting the growth of dynamic and static *S. aureus* biofilms on various surfaces, including silicone catheters, siliconized glass, and silicone-coated latex catheters. Effective immobilization reliability on solid support surfaces.	Yang et al. (2021)
LyS15S6	*Salmonella*	High enzymatic activity. Broad lytic spectrum against *E. coli, Shigella, P. aeruginosa, A. baumannii, and Klebsiella*. Good thermostability. Strong bactericidal activity against *Salmonella*. 1 μg/mL EPL (ɛ-poly-L-lysine), 2 μM LyS15S6 can make 3–4 log viable cell reductions after 2 h incubation at 25°C of the tested Enterobacteriaceae. 2.56 and 3.14 log reductions of *Salmonella* after 15 min of incubation at 25°C and 2 h of incubation at 8°C, respectively.	Han et al. (2019)
LysPBC1	*Bacillus cereus*	Rapidly kills the *B. cereus* host bacteria. Broad host specificity. Biocontrol agent against *B. cereus*. lyse all *B. cereus* group bacteria, including *B. cereus, B. weihenstephanensis, B. mycoides, B. weihenstephanensis* and *B. thuringiensis.*	Kong and Ryu (2015)
Psa and Psm	*C. perfringens*	Psa has high lytic bactericidal activity against *C. perfringens*. Good synergistic activity of 1.25 μg/mL of Psa and 3.9 0.16 μg/mL Psm. Psa and Psm together are helpful in the treatment and prevention of *C. perfringens* infections.	Sekiya et al. (2021)
LysCPS2	*C. perfringens*	Broad-spectrum antimicrobial activity. Highly specific against the strains of *C. perfringens*. High thermostable endolysin. Retains 30% of its catalytic activity against *C. perfringens* after 10 min of reaction time at 95°C. Best bactericidal activity at pH 7.5–10 and temperature 25–65°C. Highly stable in a wide range of concentrations of NaCl. Detection and biocontrol agent against *C. perfringens*.	Ha et al. (2018)
LysSE24	Multidrug-Resistant *Salmonella Strains*	Broad-spectrum activity against 23 multidrug-resistant *Salmonella* strains. LysSE24 is relatively stable at pH levels of 4.0 to 10.0 and temperatures of 20 to 60°C. 0.1 μM LysSE24 for up to 5 min reaction time denatured the *Salmonella Enteritidis*.	Ding et al. (2020)
LysSP1	*S. typhimurium*	Good bactericidal activity against both Gram-positive and Gram-negative food-borne bacterial pathogens. 10 μg of LysSP1 combined with EDTA can kill all 10^6^ CFU/mL of *Salmonella* cells. Showed lytic activity against salmonella strains*, E. coli, E. coli O157, S. aureus, L. monocytogenes* and *Shigella.* Best activity at 40°C. Stable at 4°C for 7 days and 180 days at-20°C. Active against Gram-negative and Gram-positive bacterial strains.	Jiang et al. (2021)
LysSTG2	*S. typhimurium*	High thermal stability. 93% lytic activity after heating at 50°C. 100 μg/mL against *S. typhimurium* NBRC 12529 planktonic cells and its biofilms showed a 1.2 log reduction after 1-h incubation. 40 mg/L of chlorine and 100 g/mL of LysSTG2 eliminated more than 99% of biofilm cells.	Zhang et al. (2021)
LysT144	*S. typhimurium*	Wide broad-spectrum activity against *Salmonella*. 2 μg/mL has a fast and significant lytic activity against *S. typhimurium*. Within 30 min reaction time, reduced the OD600nm of chloroform-treated *S. typhimurium* from 0.80 to 0.14.	Yang et al. (2020b)

## 5. Current challenges

Despite their effectiveness, endolysins have significant limits in their practical application in food industries. Although there are several endolysins in nature, identifying unique and potent endolysins necessitates preliminary measures such as phage isolation, propagation, endolysin cloning, and purification (Lee et al., 2021). Certain endolysins have low expression levels and are insoluble (Son et al., 2021). Furthermore, research on endolysins as food supplements and antimicrobial agents is still in its early stages because many endolysin uses have concentrated on their preventive and therapeutic efficiency against bacterial infections in animal models (Garcia et al., 2010). Endolysins’ antibacterial efficacy varies according to the food ingredients including proteins, carbs, fat, minerals, vitamins and biochemical components which mainly include temperature, pH, and ionic strength in foodstuffs (Shannon et al., 2020). All of these factors could have an impact on the protein stability and/or function of endolysins, making them less useful. As a result, only a few scientific investigations have proved their applicability in dietary supplements (Shannon et al., 2020).

### 5.1. Stability of endolysins

The stability of endolysins used in the food is a main difficulty and concern. On a laboratory scale, natural and engineered endolysins have shown remarkable antibacterial activity. However, given that food is a massively complex of foodstuffs with distinct factors, such as ion concentration, acidity, salt concentration, temperatures, and physical properties, there might be a significant difference in their antimicrobial effectiveness in dietary settings compared to *in vitro* used (Shannon et al., 2020). The reported endolysins, LysAB54 and PlyA, showed a loss of bactericidal activity in the milk. These may be due to environmental factors like salt and different protein interactions, which can neutralize the endolysins (Yang et al., 2015b; Khan et al., 2021). Additionally, research into the structure of individual domains in endolysins, the location and characteristics of linker sections, the distribution of charges, and the use of cofactors can shed light on how endolysins interact with certain meals (Shannon et al., 2020). Novel delivery ways, such as encapsulating the endolysins into some nano-vesicles, may be attempted to synthesize Gram-negative endolysins for food safety (Gondil and Chhibber, 2021; Lee et al., 2022). At the same time, we suggest that advanced computational and structural analysis ought to serve as the foundation for endolysin designs that are more logically planned and can enhance antibacterial activity in particular foodstuffs.

### 5.2. Reduced activity of endolysins

During the stationary phase, the bacteria undergo several changes, including spherical cell growth, a hard cell envelope, intense cross-linking of the cell wall, a reduction in membrane fluidity, and the activation of the robust response mechanism to help the cells escape from the disruption (Jaishankar and Srivastava, 2017). Due to this reason, bacterial growth phases have a big effect on the activity of endolysin and endolysins show less activity against the stationary phase of bacteria. Endolysin can be combined with other antibacterial agents to achieve a synergistic effect and improve lysis activity. The engineering of endolysin with the outer membrane-permeabilizing polypeptide, chitosan nanoparticle, and other LPS disrupter molecules including EDTA, and citric acid (Murray et al., 2021; Kocot et al., 2023). Also, we suggest the combination of different endolysins can be used in synergism to enhance the activity of endolysin against the stationary phase of bacteria.

### 5.3. Challenge against Gram-negative bacteria

It has been discovered that several endolysins function as broad-spectrum antibacterial agents against Gram-positive bacteria (Yang et al., 2015a; Huang et al., 2020; Yang H. et al., 2020). Contrarily, a protective bacterial outer membrane renders most endolysins ineffective against Gram-negative bacteria (Abdelrahman et al., 2021; Khan et al., 2021). Recently, bacteriophage and endolysin encapsulation methods have been reported (Loh et al., 2021). Developing novel phage and endolysin delivery methods and their application are alternative strategies to overcome endolysins’ challenges and improve endolysins’ efficiency (Elkhoury et al., 2020). Therefore, through this strategy, the endolysins can interact with the peptidoglycan layer directly (Oliveira et al., 2012; Deshmukh et al., 2021). Moreover, Yves Briers described novel ways to disrupt the Gram-negative bacterial membrane: (1) physical or chemical methods to disrupt the outer membrane’s integrity; and (2) protein engineering to provide endolysins with the tools needed to overcome the outer membrane (Gutiérrez and Briers, 2021). Another method for combating Gram-negative pathogens is to fuse endolysin with a peptide with outer membrane-permeabilizing features to the endolysin. By attaching the peptide cecropin A to LysMK34 to its N terminus via a linker consisting of three Ala-Gly repeats, resulting in a genetically engineered LysMK34 (eLysMK34). Compared to the parental endolysin, the modified engineered endolysin shows improved antibacterial activity (Abdelkader et al., 2022). To boost outer membrane permeability and increase activity against Gram-negative bacteria, use endolysin in combination with antibiotics. LNT113, an engineered endolysin, was evaluated for synergistic effects with standard-of-care antibiotics. The LNT113 showed a synergistic activity with antibiotics (Hong et al., 2022). Hurdle technology: The use of hurdle technology provides a strategy for enhancing the efficiency of endolysins against Gram-negative bacteria. The makes use of multiple methods combined to eradicate bacteria and ensure the microbiological safety of food. In the case of endolysin, the procedure requires the use of another treatment first, which is to break cell membranes, including the outer membrane allowing the lysin to interact with the target petidoglycan (Oliveira et al., 2012; Mukhopadhyay and Gorris, 2014). The further strategies to increase the bactericidal activity of endolysin against gram-negative bacteria are the development of engineered endolysin. The methods of engineering of endolysin include the development of Lysocins, Innolysins, Lysin-based dendrimers, *In silico* search for antimicrobial peptides, Liposome-mediated lysin delivery, encapsulation in alginate-chitosan nanoparticles (Kocot et al., 2023).

### 5.4. The expensive production of endolysin

**Figure 4:**
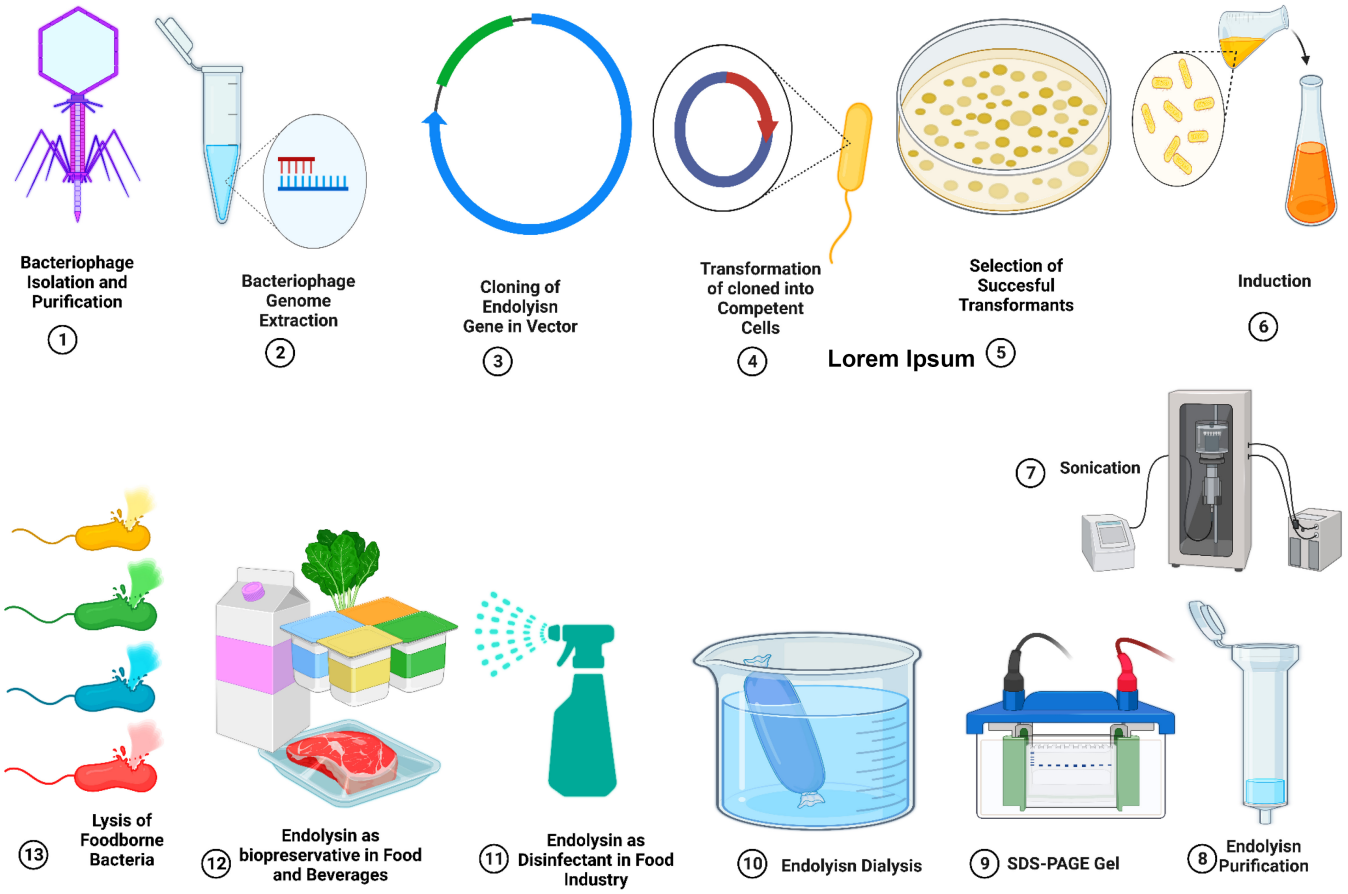
Cloning, expression, and purification of bacteriophage-derived endolysins. Applications of endolysin as a natural bio-preservative agent to reduce the contamination of foods from food-borne pathogenic bacteria.

Endolysins can eradicate Gram-positive bacteria has been proven in food matrices and specific food products, which strongly suggests that they could be used to fight foodborne pathogens in the food industry. However, the necessity for their large-scale production, purifying procedure, and scalability, which may include expensive costs, limits the use of endolysin in food companies (Xu, 2021). We should carefully assess the safety, stability, and manufacturing costs of engineered endolysins before using them in the food sector. Therefore, to address the remaining issues with endolysin, ongoing research into related issues is necessary due to the high needs of the food sector (Lee et al., 2022). Figure 4 shows the endolysin purification process.

## 6. The synthetic engineering of endolysin

Numerous approaches to synthesizing engineering endolysins can overcome the primary limitations of natural endolysins: (1) low bactericidal efficacy and a limited host range in the presence of food ingredients, and (2) lack of capacity to effectively kill Gram-negative bacteria. Endolysins’ low catalytic effectiveness and narrow host spectrum could be modified depending on the feature of endolysins, “modularity.” The modular architecture of general Gram-positive endolysins is made up of various EADs and CBD (Gerstmans et al., 2018). Their active domain units can provide simple and efficient engineering tools such as (i) direct genetic mutations (ii) domain deletion or truncation and (iii) domain shifting (Lee et al., 2022).

### 6.1. Improving the activity of endolysin through domain truncation or deletion

Endolysins’ antibacterial activity, CBD dependency, and host specificity can all be altered by the truncation or deletion of a domain (Cheng and Fischetti, 2007). The *Streptococcus* endolysin PlyGBS were randomly mutated. The mutated endolysin had about 20-fold higher lytic activity than full-length PlyGBS. Horgan et al. (2009) synthesized several variants of the *Staphylococcus* LysK endolysin and reported that CHAPk (a truncated LysK-engineered endolysin) had better lytic activity against *staphylococcal* strains *in vitro* than the natural. Low et al. (2011) revealed that eliminating CBD from several endolysins improved their enzymatic activity. They explained this phenomenon in the context of an EAD domain’s CBD dependability being closely linked to its charge. Because lipoteichoic acids are negatively charged in the cell wall, changing the net charge of the catalytic domain from negative to positive may result in enhanced interaction between the catalytic domain and lipoteichoic acids.

### 6.2. Enhancing the activity of endolysin through direct mutagenesis

The direct mutation was used further to improve the endolysins’ lytic activity and thermostability. Direct mutation in conserved sites involved in hydrogen bond networking at the backside of the amidase domain’s catalytic sites resulted in higher lytic activity of mutant CD27L endolysin against multiple *L. monocytogenes* serotypes (Mayer et al., 2011). Díez-Martínez et al. (2013) synthesized Cpl-7S, a synthetic endolysin, by swapping 15 amino acids into CBD. The alteration enhanced CBD’s net charge, resulting in improved lytic activity. For the changeable sites within the LysF1 fold, Love et al. (2021) altered a single residue in a non-conserved hydrophobic core to increase its heat stability. Using site-directed mutagenesis based on protein structure may be an appropriate approach for enhancing endolysins’ functions.

### 6.3. Domain shifting approach

Endolysin domains can act independently, they can be replaced or recombined with domains from other endolysins to develop chimeric enzymes with desirable features. Indeed, multiple studies have shown that these tactics are effective in enhancing catalytic efficiency (Mao et al., 2013; Yang et al., 2014b; Díez-Martínez et al., 2015). And increasing host specificity through CBD shifting or addition (Yoong et al., 2004; Becker et al., 2009; Zhou et al., 2017). Fusion endolysins with improved lytic activity and thermostability have been effectively tested on milk and poultry (Rodríguez-Rubio et al., 2012). Rodrguez-Rubio et al. reported that the fusion protein CHAPSH3b which is composed of the CHAP domain of HydH5 and the SH3b domain of lysostaphin eradicated *S. aureus* in pasteurized milk to undetectable numbers after 15 min of treatment at a relatively low concentration of 1 μM (Rodríguez-Rubio et al., 2012). Mao et al. (2013) increased the lytic activity of Ply187 in milk through the development of a fusion protein CBD of Lysk (KSH3b) and the EAD of Ply187. Using high-throughput synthesis and screening of chimeric endolysins, a random domain-swapping approach coupled with domain shuffling has been developed. Yang et al. (2015a) developed an *in vitro* screening platform using a two-vector *E. coli* expression system. Using the *in vitro* screening platform, they reported ClyR, a new chimeric endolysin that showed a wide, evident lysis zone in the *Streptococcus dysgalactiae* lawn. In pasteurized milk, ClyR demonstrated a wider host range and lytic activity against *Streptococcus* spp. Lee et al. (2021) developed a library of chimeric endolysins by rearranging the domains of 12 native staphylococcal endolysins. ClyC, the most efficient chimeric endolysin, maintained its significant antibacterial action in milk, and animal blood, as well as protecting against MRSA-induced bacterial infection in an *in vivo* model of mouse infection.

### 6.4. Engineering endolysins for the biological control of Gram-negative bacteria

Since the cell lysis of Gram-negative bacteria through natural endolysin therapy is limited, the basic objective of endolysin engineering for targeting Gram-negative bacteria is to enter the outer membrane (Oliveira et al., 2012). Endolysins targeting Gram-negative bacteria have previously been proven and showed synergistic activities through physical hydrostatic pressure (Nakimbugwe et al., 2006). Gram-negative endolysin was also reported which showed synergistic activities in combination with citric acid, chloroform, EDTA, Triton X-100, trichloroethane (CHCL3) (Briers et al., 2014; Yang et al., 2015b; Guo et al., 2017).

#### 6.4.1. Artilysins

Briers et al. (2014) developed the name “artilysins, “which are chimeric endolysins paired with Gram-negative lysins and an OM-destabilizing peptide. To destabilize the negatively charged lipopolysaccharides at the outer membrane, peptides with various physicochemical properties including cationic, hydrophobic, or amphipathic can be used in the engineering of artilysins. Art-175 is a representative of artilysin, which is a fusion protein composed of a modified form of endolysin KZ144 and SMAP-29. Art-175 showed antibacterial properties against a broad spectrum of Gram-negative bacteria (Briers et al., 2014).

#### 6.4.2. Lysocin and Innolysin

The gram-negative endolysins can be engineered by making use of colicin-like bacteriocins, which function in translocating the OM of bacteria and delivering endolysins to the peptidoglycan (Lukacik et al., 2012; Yan et al., 2017; Heselpoth et al., 2019). Lysocins are fusion proteins that are composed of endolysin and bacteriocin that help transport molecules over the outer membrane. Additionally, a unique strategy for targeting Gram-negative bacteria is the fusion of bacteriophage receptor binding proteins (RBPs) with endolysins, known as Innolysin (Zampara et al., 2020). Innolysin Ec21 showed potential antibacterial activity, resulting in over a 3-log decrease in *E. coli* cells resistant to third-generation cephalosporins (Zampara et al., 2020).

#### 6.4.3. Encapsulation of endolysin

Despite phages and endolysins’ antibacterial potential, these alternative agents need to overcome various practical obstacles posed by the host system, such as limited bioavailability loss of action, non-targeted delivery, fast clearance by the reticuloendothelial system, and antibody-mediated deactivation (Loh et al., 2021). A formulation approach to Gram-negative endolysins has been engineered to increase its permeability in addition to the fusion engineering method (Bai et al., 2019). Bai et al. (2019) used the cationic liposomes to encapsulate the *Salmonella* endolysin BSP16Lys. In *S. typhimurium* and *E. coli* cells, encapsulated BSPLys revealed improved antimicrobial activity of up to 2.2 logs and 1.6 logs CFU/mL decreases, respectively. T4 lysozyme was immobilized to cellulose nanocrystals, and this immobilization technique significantly improved the antibacterial activity against *E. coli* and *Pseudomonas mendocina* compared to the natural enzyme (Abouhmad et al., 2017). As the pneumocidal activity of endolysin, Cpl-1 is retained in chitosan nanoparticles. The alternative encapsulating technique using non-biohazardous and biocompatible particles, such as chitosan, can be used to synthesize Gram-negative endolysins for food safety (Gondil et al., 2020a).

## 7. Conclusion

Various foodborne outbreaks have persistently put at risk food safety despite developments in modern technology. This threat has increased as a result of antibiotic overuse, abuse, and misuse due to the increase of multidrug-resistant bacteria. To preserve the safety of the food, researchers must develop new techniques for reducing microbial contamination. Bacteriophage-derived endolysins have been suggested as innovative, efficient, and safe antimicrobial agents and applied for the control and eradication of bacterial contaminants in foods and food processing companies. Most bacteria prevalent in the food industry are Gram-negative bacteria. Therefore, the development of new antibacterials should target this group of bacteria. The bacteriophage-derived endolysin has been extensively tested for use as antibacterials. The use of endolysin in a food context is largely limited to Gram-positive bacteria, with only a few studies focusing on the use of phage lysins against foodborne Gram-negative pathogens in food matrices. In recent years, different strategies to improve the effectiveness of endolysin against Gram-negative bacteria have been reported and were focused on clinical application. Therefore, future research should focus on the control of persistent foodborne pathogens. Furthermore, endolysins have some limitations, so new endolysins can be engineered more effectively by being specially adapted for a particular food to increase lysis potential. The use of advanced molecular biology tools will help in engineering new endolysins with high activity against Gram-negative bacteria. This new and enhanced endolysin may allow us to overcome existing barriers and make endolysin used for the control of food-borne pathogens more accessible. To produce an “ideal” engineer endolysin that maintains high activity while exhibiting other desirable properties depending on the conditions of its employment. An engineer endolysin might have thermostability, and high solubility to make it accessible, highly targeted, and broad-spectrum activity depending on the target pathogen, and the production should be cost-effective. Endolysin engineering opens up a plethora of possibilities for the production of ideal novel endolysin. The development of next-generation endolysin can be applied in the food sector.

## Author contributions

FK: Conceptualization, Funding acquisition, Investigation, Writing – original draft. J-HC: Conceptualization, Formal analysis, Supervision, Writing – review & editing. RZ: Conceptualization, Supervision, Writing – review & editing, Funding acquisition, Project administration. BL: Conceptualization, Funding acquisition, Supervision, Writing – review & editing, Formal analysis, Project administration.
